# Relapse of retinal vasculitis after a non-medical biosimilar switch: a case report

**DOI:** 10.1186/s12348-026-00575-0

**Published:** 2026-04-14

**Authors:** Georges AbouKasm, Charles Zhang, Thomas A. Albini

**Affiliations:** 1 Miller School of Medicine, University of Miami, Miami, FL USA; 2 Department of Ophthalmology, Bascom Palmer Eye Institute, University of Miami Miller School of Medicine, 900 NW 17th Street, Miami, FL 33136 USA

**Keywords:** Biosimilar-to-biosimilar switch, Infliximab, Uveitis, Retinal vasculitis, Immunogenicity, Biologic therapy, Anti-drug antibodies

## Abstract

**Purpose:**

To describe a case of recurrent ocular inflammation following a non-medical switch between two infliximab biosimilars, highlighting potential clinical implications of biosimilar-to-biosimilar transitions in uveitis management.

**Case description:**

A 56-year-old man with intermediate uveitis and retinal vasculitis achieved complete remission on adalimumab but developed anti-drug antibodies after 22 months, prompting transition to infliximab-dyyb (Inflectra) with oral methotrexate. The patient achieved quiescence for two years on this regimen. Due to an insurance formulary change, therapy was switched to another infliximab biosimilar, infliximab-axxq (Avsola), at the same dosage (5 mg/kg every 8 weeks). Within two months, he experienced recurrent vitreous inflammation and vasculitis confirmed by fluorescein angiography. Following insurer approval, he was reverted to infliximab-dyyb, resulting in prompt resolution of inflammation within one infusion cycle. He has since remained in remission for three years on infliximab-dyyb, with sustained 20/20 visual acuity bilaterally.

**Conclusion and importance:**

This report describes, to our knowledge, the first case of a temporal association between a biosimilar-to-biosimilar switch and disease relapse in a single patient with ocular inflammatory disease. While biosimilars undergo rigorous regulatory testing to confirm clinical equivalence to the reference product, subtle molecular or formulation variations, such as glycosylation or excipient differences, may influence immunogenicity and drug bioavailability in individual patients. As anti-VEGF biosimilars enter into ophthalmic use, clinicians should exercise vigilance during mandated switches, ensuring close monitoring for relapse. Individualized management and open insurer communication are critical to maintaining disease control and optimizing patient outcomes.

## Introduction

Biologic therapies have transformed the treatment of many diseases, and biosimilars aim to expand access to these medications by lowering costs. A biosimilar is a medication that is highly similar to an already FDA-approved biologic; however, unlike a traditional generic drug, which are identical small-molecule chemicals, biosimilars are not an exact copy of their reference drug [1]. Biosimilars possess minor differences in the molecular structure (such as glycosylation) but are found to have no clinically meaningful differences in safety, purity or potency compared to the reference product [1]. Clinical trials and real-world studies across rheumatology, oncology and other fields of medicine have confirmed that approved biosimilars produce equivalent therapeutic outcomes to their originator biologics [2–5]. 

Ophthalmology is now entering the biosimilar era. In 2021, the first ranibizumab biosimilars were approved in the US. Retina specialists so far have been cautious in adopting biosimilars [6]. This hesitancy is not due to demonstrated efficacy, but rather due to the availability of off-label bevacizumab which is available at a much lower cost and large clinical trials demonstrating no difference in efficacy of bevacizumab as compared to ranibizumab [6, 7]. Since 2024, multiple aflibercept biosimilars are poised to or have entered the market [8]. Given the large population of patients receiving aflibercept therapy and the potential cost benefits of biosimilars, insurers may mandate switching to biosimilars for non-medical reasons.

While biosimilars are intended to be interchangeable in effect, clinicians have raised concerns that even subtle molecular or formulation differences might impact individual patients [9]. There have been reports in the literature suggesting that not all patients maintain the same level of disease control after a switch [10]. Multiple reports exist in the literature of uveitis patients who developed flares within 2–3 months after switching from original reference immunosuppressants to a biosimilar after long periods of remission on the reference product [11–13]. The current report highlights how this type of event can occur even when switching from one biosimilar to another. To our knowledge, this is the first report documenting a differential clinical response between two biosimilars referencing the same biologic in the management of ocular inflammatory disease.

## Case report

**Fig. 1:**
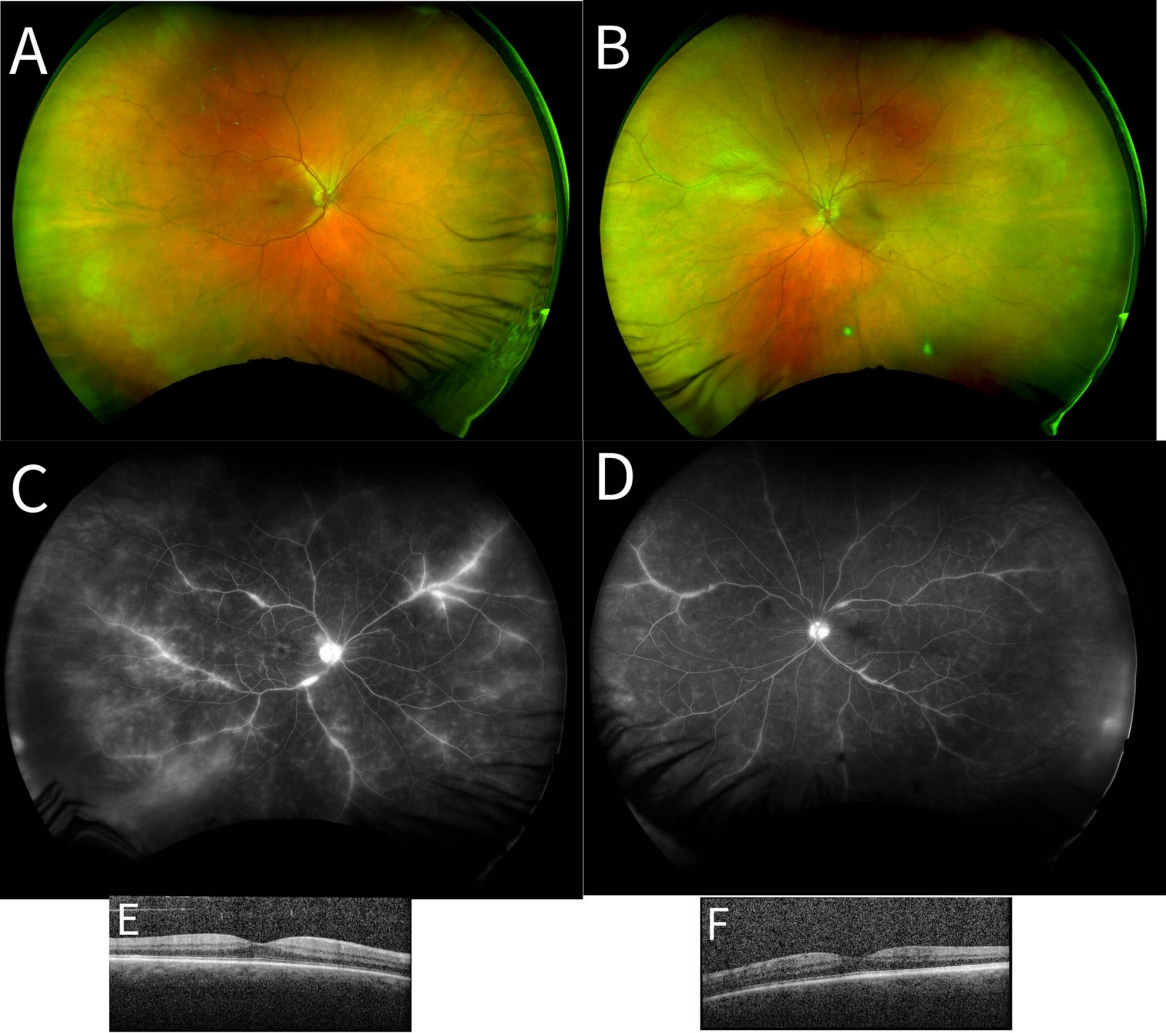
Patient at initial presentation of vasculitis. **A**. Fundus photo of the right eye. **B**. Fundus photo of the left eye. **C**. Fluorescein angiography of the right eye. **D**. Fluorescein angiography of the left eye. **E**. OCT of the right eye. **F**. OCT of the left eye

A 56-year-old male with a history of psoriasis (inactive, not on systemic treatment) was referred to uveitis clinic for evaluation of intermediate uveitis with retinal vasculitis. At presentation, his best-corrected visual acuity (BCVA) was 20/40 in the right eye (OD) and 20/30 in the left eye (OS). Intraocular pressures (IOP) were 12 mmHg OD and 13 mmHg OS. Anterior segment exam showed trace ciliary flush, but no anterior chamber cells. Vitreous exam revealed 2 + vitreous cells with clusters of inferior snowballs, and there was prominent perivenular sheathing of the retinal veins, consistent with vasculitis (Fig. 1A/B). Fluorescein angiography (FA) demonstrated perivenular leakage (staining) extending from the posterior pole to the periphery (zones 1–3), significant optic disc leakage, and mild petaloid leakage in the macula OD > OS (Fig. 1C/D). Optical coherence tomography (OCT) of the macula showed vitreous opacities but no evidence of cystoid macular edema (Fig. 1E/F). A comprehensive systemic workup for underlying inflammatory or infectious etiologies was unrevealing. Notably, tests for tuberculosis, syphilis, rheumatoid factor, antinuclear antibody, anti–double stranded DNA, ANCA, angiotensin-converting enzyme, and lysozyme were all within normal limits or negative. HLA-B27 was negative. A chest X-ray was normal, and an MRI of the brain showed no signs of cerebral vasculitis or demyelination.

The patient was started on oral prednisone 20 mg daily along with adalimumab 40 mg subcutaneously every 2 weeks. At the 3-month follow-up visit, the patient’s BCVA had improved to 20/20 in both eyes with significant improvement of both the vitreous opacities and resolution of the retinal vasculitis on FA. The prednisone was gradually tapered off over the ensuing 2 months, and the patient was maintained on biweekly adalimumab monotherapy. He remained quiescent on adalimumab for nearly two years.

**Fig. 2:**
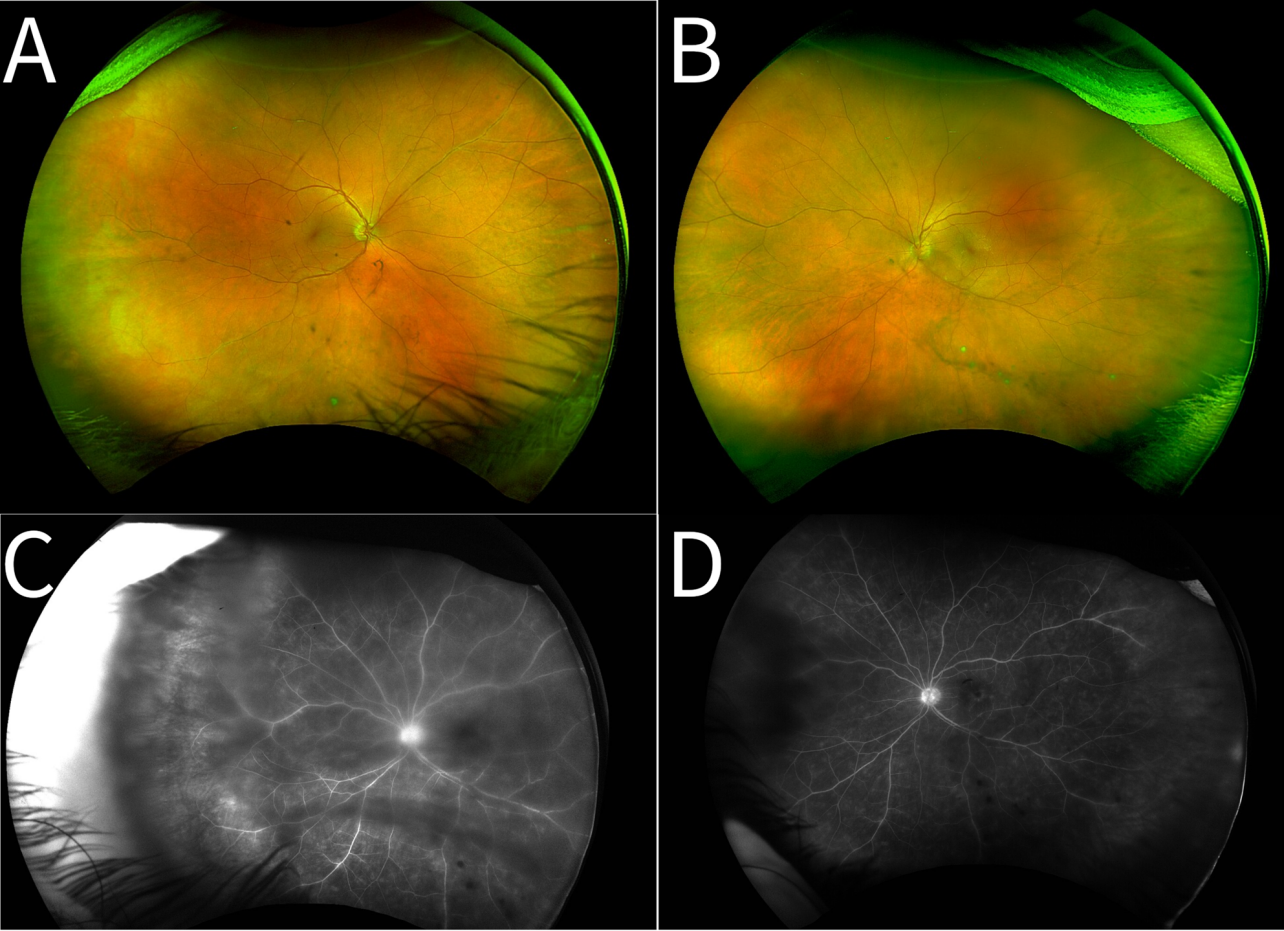
Patient flare up at approximately 22 months after starting adalimumab. **A**. Fundus photo of the right eye. **B**. Fundus photo of the left eye. **C**. Fluorescein angiography of the right eye. **D**. Fluorescein angiography of the left eye

Approximately 22 months after starting adalimumab, the patient noted new onset floaters, OS > OD. On examination, VA was 20/20 OD and 20/25 OS. Anterior segment exam remained quiet, but there was a recurrence of vitritis with 1 + vitreous cells OD and 2 + OS, along with new inferior snowballs (Fig. 2A/B). FA confirmed worsening retinal periphlebitis (Fig. 2C/D). Concerned for a possible loss of response to adalimumab, anti-adalimumab antibody levels were obtained that was found to be elevated at 274 ng/mL (normal < 25 ng/mL). Given this loss of efficacy, the decision was made to switch therapy to infliximab in combination with oral methotrexate to reduce further antibody formation. Per insurance requirements, the patient’s infliximab was initiated not as the Remicade brand, but as an infliximab biosimilar product called infliximab-dyyb (marketed as Inflectra). He received a standard induction and maintenance regimen (5 mg/kg IV infusions at 0, 2, and 6 weeks, then every 8 weeks). Methotrexate 17.5 mg weekly by mouth was given concurrently. The switch to infliximab-dyyb, along with methotrexate, achieved an excellent response. Over the next 3 months, the vitreous inflammation resolved, and follow-up FA showed no active vasculitis. The patient continued on infliximab-dyyb infusions every 8 weeks with methotrexate and remained stable and inflammation-free for 24 months, with serial fluorescein angiography performed approximately every 6 months confirming absence of vascular leakage or active vasculitis.

**Fig. 3:**
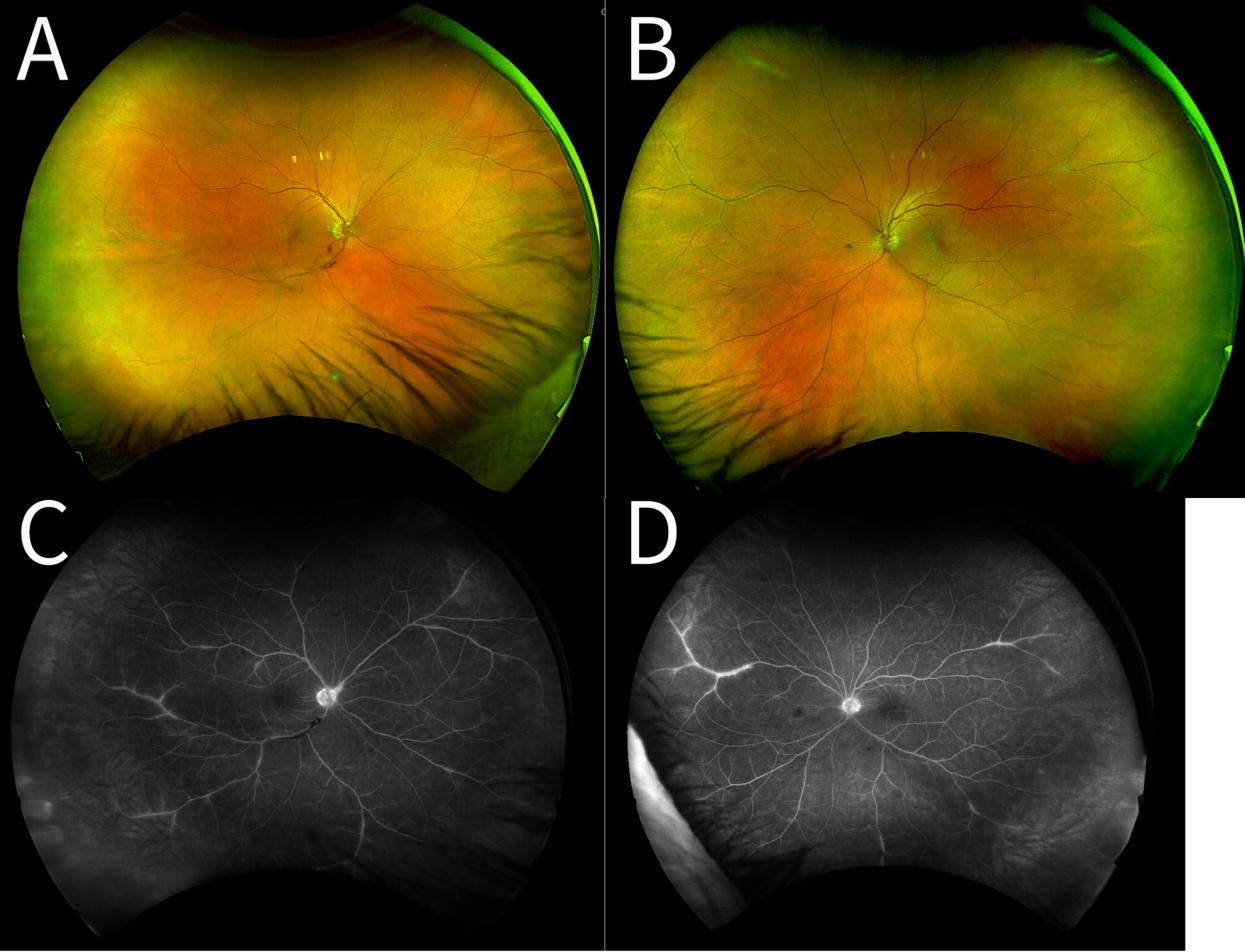
Patient presentation at approximately 2 years after starting infliximab-dyyb and before switching to infliximab-axxq. **A**. Fundus photo of the right eye. **B**. Fundus photo of the left eye. **C**. Fluorescein angiography of the right eye. **D**. Fluorescein angiography of the left eye

**Fig. 4:**
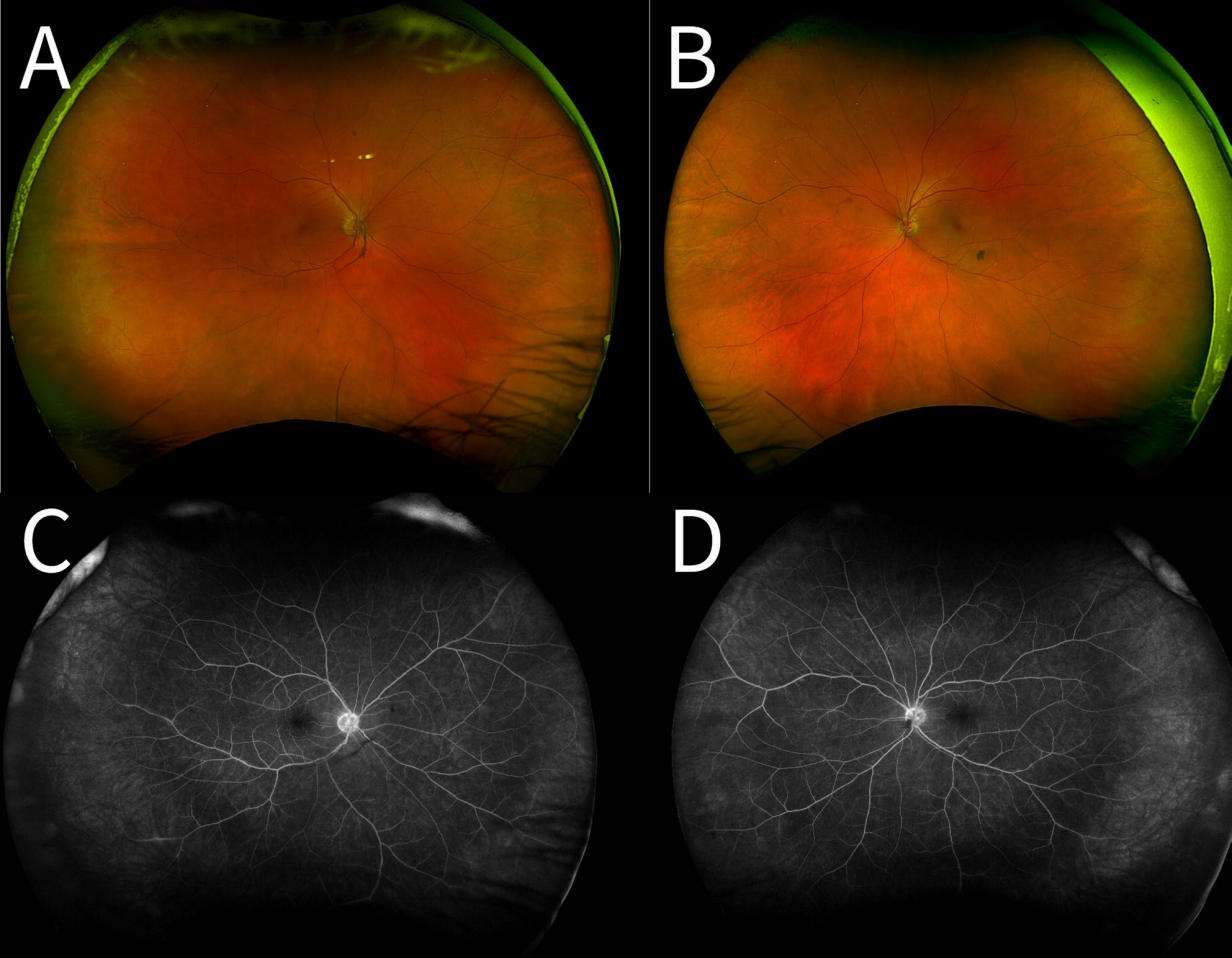
Patient presentation at last follow up following switch from infliximab-axxq back to infliximab-dyyb at approximately 36 months of resuming infliximab-dyyb. **A**. Fundus photo of the right eye. **B**. Fundus photo of the left eye. **C**. Fluorescein angiography of the right eye. **D**. Fluorescein angiography of the left eye

Approximately 2 years after starting infliximab-dyyb, a non-medical change was made to his treatment. The patient was switched to infliximab-axxq (Avsola) with the same dosing due (5 mg/kg every 8 weeks) to a new insurance formulary policy. Two months after transitioning to infliximab-axxq, the patient returned with complaints of increased floaters again. Visual acuity was still 20/20 in both eyes, and anterior segments were quiet, but dilated exam revealed a reappearance of vitreous cell and vasculitis (Fig. 3A/B), that was confirmed on FA (Figs. 3 C/D). After discussion with the patient and accumulation of photographic evidence of the flare, a petition was made to the insurance company for a return to the previous infliximab biosimilar (infliximab-dyyb) on the grounds of medical necessity. After completing two 8-week dosing intervals on infliximab-axxq and receiving insurance approval, the patient was switched back to infliximab-dyyb at his next scheduled infusion. Within one infusion cycle of reverting to infliximab-dyyb, the retinal vasculitis again quieted down. At his last follow-up (now 36 months since resuming infliximab-dyyb, for a total of 5 years on infliximab therapy), the patient has remained in complete remission with no active vasculitis (Fig. 4A-D). His visual acuity remains 20/20 OU.

## Discussion

The current report describes a real-world scenario in which a patient experienced disease relapse temporally associated with switching between two infliximab biosimilars. The patient’s retinal vasculitis was well-controlled on an infliximab biosimilar (infliximab-dyyb), but experienced reactivation of disease when forced to a different infliximab biosimilar (infliximab-axxq). Restoration of the original biosimilar led to renewed control suggesting it was the drug switch was the source of the reactivation. To our knowledge, this is the first reported case in ophthalmology of a biosimilar-to-biosimilar switch associated with a loss of efficacy.

From a regulatory and clinical trial perspective, all biosimilars of a given reference are expected to be interchangeable in outcome [1]. In fact, multiple studies in rheumatologic and gastrointestinal diseases have shown that patients can be switched from a reference biologic to a biosimilar (and even between different biosimilars) without loss of efficacy or new safety issues in the vast majority of cases [14–17]. A recent systematic review identified 90 studies on over 14,000 patients examining reference product-to-biosimilar switching, and concluded that such switches were generally safe, effective, and did not significantly affect clinical outcomes in 14 different diseases [18]. Additionally, one study on inflammatory arthritis evaluated patients who switched from one adalimumab biosimilar (ABP501) to another (SB5) led to no significant changes in disease activity with remission rates and drug survival remaining high [19]. Similarly, within ophthalmology, many large series exist in literature that demonstrate patients switching from reference biologic immunosuppressants had similar levels of clinical disease activity post-switch [20, 21]. However, while these studies demonstrate that as an aggregate most individuals do well on these switches, a small subset may not have favorable responses [13]. 

Despite reassuring data from large switching studies, several reports in rheumatology and autoimmune disease literature have described inflammatory flares temporally associated with non-medical biosimilar transitions. Case reports and cohort studies have documented loss of disease control or subjective worsening in a subset of patients after switching biologics, including patients with Behçet’s disease and inflammatory arthritis [13, 22, 23]. These findings suggest that individual variability in immunogenic or pharmacologic response may play a role. While most patients tolerate biosimilar switching well, rare exceptions exist and warrant careful clinical monitoring. Our case extends this concept to ocular inflammatory disease.

One possibility to explain these changes could be immunogenicity. As seen in our patient, all therapeutic antibodies can induce anti-drug antibodies (ADA), especially in chimeric proteins like infliximab [24]. The patient described in this report had already developed ADA to adalimumab and was started on methotrexate to reduce the rate of ADA formation against the new infliximab medication. However, it is conceivable that minor structural or formulation differences in the second biosimilar (infliximab-axxq) might have triggered a new immune response that could reduce the drug’s bioavailability. Studies have noted that different infliximab biosimilars have slightly difference glycosylation profiles, which could potentially affect their interaction with immune cell or even clearance rates [25]. One report of 3 patients with Behcet’s disease that developed relapse of disease after switching from reference infliximab to an infliximab biosimilar was postulated to occur due to cross-reactive ADAs that might not have bound significantly to the reference infliximab but did neutralize the biosimilar [13]. 

Another consideration is pharmacodynamics or potency. Although biosimilars meet rigorous equivalence standards, the manufacturing process can result in small difference in the molecular structure of each drug. These could translate into slight differences in binding affinities or half-lives. If a patient is on the precipice of disease control, even a minuscule drop in effective drug concentration could permit inflammation to break through. One series found that patients who flared after switching to infliximab-abda tended to require slightly higher doses to regain quiescence, implying the biosimilar’s effective activity in those individuals might have been marginally lower [11]. It is worth noting that in this study, 2/3s of patients who flared did so within 3 months of the switch which is on a similar timeline to the current report.

It is important to note that these observations do not necessarily suggest that biosimilars are inferior products, but rather that each biosimilar may exhibit subtle pharmacologic or immunologic differences that can lead to differences in efficacy on an individual level. The current case underscores the opposite scenario where the patient experienced decreased efficacy of reference adalimumab due to ADA formation and was switched to a biosimilar to bypass the immunologic blockage. Thus, rather than a binary view of reference versus biosimilar quality, it may be more accurate to recognize that each biologic, both reference and biosimilar, represents a unique molecular entity whose performance can vary across individuals. As a single case report, this observation does not establish comparative efficacy between infliximab biosimilars. Rather, it highlights a temporal association between a non-medical switch and disease relapse in one patient and suggests the possibility of interindividual variability following biosimilar transitions. Consequently, any transition (reference-to-biosimilar, biosimilar-to-biosimilar, or biosimilar-to-reference) carries a potential, albeit small, risk of disease relapse due to subtle molecular or immunogenic differences.

As retina specialists begin to use anti-VEGF biosimilars for AMD and other retinal conditions, it will be crucial to remain vigilant during therapy transitions. Anti-VEGF biosimilars have shown equivalent efficacy in clinical trials for AMD, and no significant safety issues have emerged in the early adopters [26]. However, if an insurance company mandates switching a well-controlled AMD patient from one drug to another, one must consider the potential risk of a lapse in disease control. In neovascular AMD, even a short period of renewed exudation can cause irreversible damage to the retina [27]. Recurrent fluid, hemorrhages, or scarring from a delay or loss of efficacy could translate into loss of visual acuity that cannot be fully recovered. The cost savings from biosimilars will only be meaningful if outcomes remain equivalent to the reference drugs; a single preventable vision loss event could nullify those benefits from a patient perspective.

In summary, this case emphasizes that “biosimilar” does not always equate to “identical” in real-world clinical practice for every patient. Biosimilars remain rigorously tested, valuable options that will improve access to sight-saving therapies in retinal disease. However, clinicians should remain alert to the possibility of variability in individual responses, especially when switching for non-medical reasons. Should a switch be necessary, proactive monitoring is needed to ensure that patients continue to receive the best possible medication for their disease.

## Data Availability

All data supporting the findings of this study are included in the article and its supplementary materials.

## Abbreviations

OD: Right eye
OS: Left eye
BCVA: Best-corrected visual acuity
IOP: Intraocular Pressure
FA: Fluorescein angiography
OCT: Optical coherence tomography
ADA: Anti-drug antibodies
